# Analysis of factors affecting medical personnel seeking employment at primary health care institutions: developing human resources for primary health care

**DOI:** 10.1186/s12939-022-01638-z

**Published:** 2022-03-17

**Authors:** Huanhuan Jia, Xihe Yu, Hairui Jiang, Jianxing Yu, Peng Cao, Shang Gao, Panpan Shang, Bayuzhen Qiang

**Affiliations:** 1 School of Public Health, Jilin University, Changchun City, Jilin Province China; 2 Lhasa People’s Hospital, Lhasa City, Tibet Autonomous Region China

**Keywords:** Primary health care, Medical personnel, Community health workers, Human resources development, China

## Abstract

**Background:**

The serious shortage of human resources for primary health care (PHC) is a common issue in health reforms worldwide. China has proposed that it is an effective way to encourage and guide qualified medical personnel to work in primary health care institutions (PHCIs). However, few studies have been conducted on the willingness and influencing factors of medical personnel to seek employment at PHCIs.

**Methods:**

Based on implicit theory and lexical approach, pre-investigation was conducted to collect the items that influence the medical personnel to seek employment at PHCIs from the perspective of guided objects. Through a three-phase investigation of 1160 doctors in 29 public hospitals in 9 cities, the items were categorized, and a structural equation model was established and verified to explore the interrelationship of influencing factors.

**Results:**

A total of 6 factors were rotated, including Sense of Gain (SG), Internal Organization Development (IOD), Remuneration and Development (RD), Condition of the City Where the PHCI Is Located (CCPL), Job Responsibilities (JR) and Family Support (FS). The results of the model showed that IOD, RD, JR and FS had a significantly positive effect on the SG. In addition, the FS, RD and JR significantly mediated the relationship between the internal and external environment of PHCIs and SG. The values of the fit index indicated an acceptable-fitting model.

**Conclusion:**

Family, remuneration, individual development, and job responsibility are closely related to the willingness of medical personnel to seek employment at PHCIs, and the internal and external environment of PHCIs is also an important factor. Therefore, the development of PHC providers can be promoted by paying attention to the family members of medical personnel, establishing a reasonable remuneration system, providing suitable development opportunities, arranging work rationally and improving the internal construction of PHCIs.

## Background

Primary health care (PHC) is an important measure to improve the equity in health [1], and it also plays a significant role in improving national health and avoiding excessive increases in national health investment [2]. At the same time, compared with healthcare systems with fewer primary care physicians, those based on a strong primary care workforce not only ensure health equity, but also achieve better outcomes and quality at lower costs [3, 4]. Therefore, increased attention has been focused on PHC, and many countries have implemented a series of policies to promote access to PHC, such as the construction of Family Health Units in Portugal [5], the implementation of the Affordable Care Act in the U.S. [6], and the creation of Rural Health Centers in India [7]. In China, PHC is carried out by primary health care institutions (PHCIs) [8], consisting mainly of community health service centers and township hospitals that provide general clinical care and basic public health services to residents.^1^ Considering the changes in the spectrum of diseases and the increase in disease burdens caused by an aging population [9], rapid urbanization [10], and behavioral changes [11], the Healthy China 2030 initiative takes PHC as a safeguard for equity in health, as well as regards the primary health system as the solution to the double burden of increases in expenditures [12] and noncommunicable diseases [13]. In addition, the hierarchical diagnosis and treatment system was implemented by the government to reduce the difficulty and high cost of seeking medical services, and PHCIs are the gatekeepers of residents’ health. Therefore, PHCIs play an irreplaceable role in protecting the health of residents, reducing the burden of disease, and improving the equity of health services. By 2020, China has 970,036 PHCIs, with 1.09 doctors and 0.75 registered nurses per 1000 population [14]. However, several studies have revealed that PHCIs are facing enormous challenges, such as unbalanced geographical distribution of PHC providers [15] and losses among primary care physicians [16]. Besides, previous studies had revealed that 21% of doctors practicing in PHCIs were neither licensed doctors nor licensed assistant doctors, with a larger proportion in less developed regions (33%) [16] in China. Only 29.5% of medical personnel in community health service centers had a bachelor’s degree or higher, while the proportion in township health centers was even lower (12.3%) [17]. As for the UK, primary care is the mainstay of the National Health Service (NHS) in the UK, and 90 % of NHS patient contact takes place within primary care. Besides, a general practitioner (GP) must undergo nearly 10 years of professional training such as college study, clinical practice, and community study, and pass the General Practitioner College examination before being a qualified general practitioner. Therefore, the construction of PHCIs and the utilization of PHC in China are far behind that in the UK. However, in recent years, there has been a relative shortage of GP in the UK, with the rate of increase lagging behind the rising demand for basic care and the growing population. Previous studies have shown that the number of GP per 100,000 people in the UK fell to 59.5 in 2013 [18] and the vacancy rate rose to 7.9% [19]. Therefore, the serious shortage of PHC providers is a common issue in health reforms worldwide. It has been noted that a robust and well-distributed primary healthcare workforce is the key to the success of any health reform [20], so human resource development is an important aspect of the structural optimization of the primary health care system and the equity in health.

To promote the human resource development of PHCIs, previous studies and the government have proposed a series of measures, such as training general practitioners [2, 21], balancing work pressure [22], improving financial remuneration, and providing more training and learning opportunities [23]. At the same time, encouraging and guiding qualified and senior medical personnel to work in PHCIs is also an effective way to improve the overall quality and efficiency of health care [24]. The government of China has tried to guide medical personnel toward employment in PHCIs, but this has not produced good results, and the problem of insufficient human resources in PHCIs has not been substantially ameliorated. After analyzing the previous studies and government measures, three reasons have been summarized to explain the failure. First, medical personnel and primary health care providers are rarely involved in decision-making, which usually involves governments and PHCIs, resulting in measures being developed only from the perspective of governments and institutions without considering the real needs of medical personnel. Second, although previous studies have explored the influence of financial and professional incentives [25], work environment, workplace, family, and partner [26] on physicians’ career decisions, few studies have comprehensively explored the influencing factors and the interrelationships of these factors from the perspective of medical personnel, which is important for the government and PHCI’s formulation of guiding measures. Third, county-level public hospitals, as the leading component of the three-level rural health service system [27], play a crucial role in ensuring the health of rural residents in China. However, previous studies did not distinguish between urban and county-level public hospitals, resulting in blurred selection boundaries of guided subjects. Therefore, on the premise of ensuring the efficient operation of the existing service system, the guided subjects were positioned as medical personnel in urban public hospitals, and the influencing factors of medical personnel seeking employment at PHCIs were explored.

Implicit theory refers to people’s views on the concept, structure, and development process of certain psychological features that develop during daily life and work, which exist in individual thoughts in a certain form and can accurately and comprehensively reflect people’s psychological performance [28]. Implicit theories profoundly influence individual cognition, motivation, and behaviors [29, 30] and may predict how people react to particular stimulation or behavior training. It has been widely applied in emotional and mental health [31], leadership [32], self-regulation [33], and wisdom [28]. The lexical approach is an important approach to implicit theory, which is based on the hypothesis that the most important individual differences in the daily transactions between people will eventually be encoded in people’s language [34, 35]. The relevant lexicographical code reflects people’s daily psychological thoughts, beliefs, values, and practices [36]. Therefore, the lexical approach can be used to collect the organization descriptors of a certain population on an organizational entity and then understand the group’s perception toward activities related to that organizational entity. The argument here is that, in the course of China’s health care reform, the implementation of the government’s policy and the changes in the work patterns have a profound impact on the work and life of medical personnel. The perceptions and perspectives on health care reform, PHC, and PHCIs exist in the minds of individuals, and this information is then encoded in their minds and language, reflecting their attitudes and views on health care reform. In this study, implicit theory and lexical approach were used to extract the lexicographical codes related to PHCI development and employment, and to explore the factors that influence the willingness of medical personnel to seek employment at PHCIs from the perspective of the guided objects.

## Materials and methods

### Participants and procedures

**Fig. 1:**
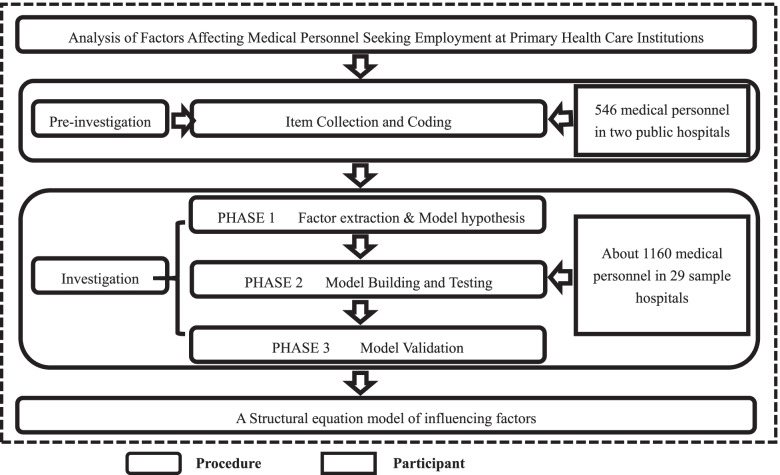
The participants and procedures in this study

According to the paradigm of lexical studies [37, 38], the research was composed of pre-investigation and investigation stages. The participants and procedures are shown in Fig. 1.

#### Pre-investigation

The purpose of pre-investigation was to collect, classify, code, and screen out the items that were of high concern to medical personnel seeking employment at PHCIs, which were the basis for the investigative questionnaire in the next stage. The participants were medical personnel in two public hospitals, and a data collection service from China’s leading online survey website was used to administer the pre-investigation.

#### Investigation

The purpose of the investigation was to explore and verify the dimensions and relationships of the items, and the participants were medical personnel in urban public hospitals. After discussing with health service and hospital management experts, and the head of the health administration department, the sample hospitals were obtained through stratified random sampling, and medical personnel were obtained through quota sampling. Specifically, by stratified random sampling, 29 hospitals were selected as sample hospitals from the urban public hospitals in Jilin province in a proportion of 1/4 according to the type and grade of hospitals. Besides, a total of 40 medical personnel, including doctors, nurses, and medical technicians, were randomly selected from each hospital at a ratio of 2:1:1 as the investigation subjects. Finally, a total of 1160 medical personnel were involved in the investigation.

The investigation consisted of three phases of on-site investigation using a paper questionnaire, and each phase was explained, collected and checked by trained investigators to ensure the integrity of the questionnaire. The purpose of the first phase was to categorize the items collected in the pre-investigation and then perform a theoretical analysis to obtain a hypothetical model of driving factors. The second phase aimed to analyze the reliability and validity of the driving factors and to establish a structural equation model. The third phase verified the stability of the structural equation model. It should be noted that the sample hospitals and sample size at the three phases were constant, whereas the personnel who were randomly selected were not necessarily the same people.

The study was conducted according to the guidelines of the Declaration of Helsinki and was approved by the Medical Ethics Committee of the authors’ institute. Following ethical standards and practices, the participants received a full explanation of the research purpose, were told that the information collected would only be used for research purposes and were made aware that they could withdraw from the study at any time.

### Measures

The pre-investigation questionnaire consisted of two parts. The first part included the sociodemographic characteristics of the medical personnel, such as gender, age, education level, profession, etc.; the second part was an open-ended question: “If a PHCI was recruiting medical personnel, what factors would influence you to seek employment at a PHCI? Please write down all these factors in simple words or phrases.” Several measures were adopted in the questionnaire design and data cleaning to ensure data quality and reduce social desirability bias. For example, personnel characteristics were identified, including whether the respondents were regular personnel or professional clinical medical personnel. Other screening questions were set up to ensure that the answers came from eligible respondents. In addition, answer time, empty item review, and IP confirmation (i.e., the same computer restriction and geographic restriction were used to prevent one respondent from filling out the questionnaire multiple times or people from other regions filling out the questionnaire) were applied to ensure the quality of the data.

In the investigation, the questionnaire consisted of two parts: sociodemographic characteristics and an evaluation of the importance of factors affecting medical personnel seeking employment at PHCIs. The factors were the items from the pre-investigation; a 5-point Likert scale was adopted to evaluate the importance of the factors, with anchors ranging from 1 (very unimportant) to 5 (very important). The question in the questionnaire was “If a PHCI was recruiting medical personnel, please evaluate the importance of the following factors and their influence on your choice to seek employment at a PHCI based on your actual situation”.

### Data analysis

The lexical data collected by the pre-investigation were cleaned and organized using the following principles without changing the meaning of the words: (a) removal of modifiers to extract key information; (b) combination of synonymous words; (c) splitting of combined concepts; (d) deletion of words that are clearly irrelevant to the research. At the same time, the items were cleaned up and organized separately by two people, and the final results were compared. The different results were summed for analysis by a panel of experts.

In the first phase of the investigation, exploratory factor analysis (EFA) was performed in SPSS software and was applied to analyze the data to obtain concise and representative factors. Principal component analysis (PCA) was performed to extract the factors from the items, and varimax rotation (VR) was used to improve the interpretability of the solution. In addition, based on these factors, a hypothesized structural equation model (SEM) was proposed. Then, for the second phase, we performed SEM following the two-step approach recommended by Anderson and Gerbing [39]. First, confirmatory factor analysis (CFA) was carried out for each factor to test whether these factors had a significant factor loading index and to analyze the reliability and validity of the questionnaire. Second, based on the hypothesized path model, the parameters were estimated by the maximum likelihood method. The bootstrap method was used to test the potential mediator effects, and we calculated the total, direct, and indirect effects. AMOS software was used for CFA and SEM. Finally, based on the constructed structural equation model, the results of the third phase were used to evaluate the stability of the model. The significance of the model was reflected by the chi-square value. However, because the chi-square value is sensitive to large sample sizes, it is necessary to check other goodness-of-fit indices [40, 41]. The following model fitting indices were tested to evaluate the model (the values in parentheses indicating the cutoffs for acceptable fit) [42, 43]: (a) the root mean square error of approximation (RMSEA ≤0.08); (b) the comparative fit index (CFI ≥ 0.90); (c) the Tucker–Lewis index (TLI ≥ 0.90); and (d) the normed fit index (NFI ≥ 0.90). and (5) the incremental fit index (IFI ≥ 0.90). All statistical tests were two-sided with the level of significance set at 0.05.

## Results

### The results of factors collection

A total of 546 personnel participated in the pre-investigation, among whom 22 people came from the preventive medicine, finance, and administrative management fields, so they were removed. Finally, 524 complete and valid responses were obtained, with an effective response rate of 95.97%. Among them, most of the medical personnel were women (74.81%), majored in clinical medicine (86.07%), and had a bachelor’s degree (67.40%); the mean age was 26.41 (± 3.07).

After lexical cleaning and organization, all lexical data was recorded into 102 items. The results showed that 55 items had a frequency of less than 4; that is, fewer than 4 (1%) of the respondents mentioned these factors, so these items were deleted due to lack of representativeness. Finally, 47 items affecting medical personnel seeking employment at PHCIs were obtained in the pre-investigation, and these items were adjusted and normalized to explore the dimensions and structure of the items in the investigation.

### Demographic characteristics of the participants in investigations

A total of 1117,1111 and 1138 valid questionnaires were collected in the three investigations. In each investigation, the majority of respondents were female and married, with college degrees and junior or middle professional titles. Most medical personnel are between the ages of 31 and 40 and have worked for between 6 and 15 years. The demographic characteristics of the subjects are shown in Table 1.

**Table 1:** Demographic characteristics

Variables	2018	2019	2020
Gender			
Male	300 (26.86)	273 (24.57)	304 (26.71)
Female	817 (73.14)	838 (75.43)	834 (73.29)
Age			
≤ 30	334 (29.90)	323 (29.07)	331 (29.09)
31–40	467 (41.81)	486 (43.74)	505 (44.38)
41–50	215 (19.25)	213 (19.17)	219 (19.24)
≥ 51	101 (9.04)	89 (8.01)	83 (7.29)
Marital status			
Unmarried	204 (18.26)	217 (19.60)	249 (21.88)
Married	878 (78.60)	855 (77.24)	854 (75.04)
Other	35 (3.13)	35 (3.16)	35 (3.08)
Education			
High school and below	46 (3.58)	39 (3.51)	37 (3.25)
Junior college	224 (20.05)	204 (18.35)	207 (18.20)
College	614 (54.97)	646 (58.09)	650 (57.12)
Master’s degree and above	240 (21.40)	223 (20.05)	244 (17.57)
Professional title			
Senior	54 (4.83)	51 (4.59)	39 (3.43)
Sub-senior	190 (17.01)	175 (15.74)	191 (16.78)
Middle	351 (31.42)	359 (32.28)	351 (30.84)
Junior	451 (40.38)	433 (38.94)	429 (37.70)
None	71 (6.36)	94 (8.45)	128 (11.25)
Years of work			
≤ 5	413 (36.97)	371 (33.39)	396 (34.80)
6–15	417 (37.33)	456 (41.04)	462 (40.60)
16–25	133 (11.91)	135 (12.15)	149 (13.09)
≥ 26	154 (13.79)	149 (13.41)	131 (11.51)
Total	1117 (100.00)	1111 (100.00)	1138 (100.00)

### Exploratory factor analysis and research hypotheses

The EFA results of the first phase of the investigation showed that the Kaiser–Meyer–Olkin (KMO) value was 0.975, higher than 0.6, and the result of Bartlett’s test was significant (*χ2* = 46,123.739, *p* < 0.001), both of which indicated a strong correlation among the items; the data were applicable for EFA [44, 45].

The first EFA result showed that a total of 6 factors with eigenvalues greater than 1 were rotated, and the cumulative contribution rate reached 69.540%. However, there were 9 items with factor loads less than 0.4 or more than 0.4 in two or more factors simultaneously, so they were gradually deleted. In the end, 5 factors were rotated from the remaining 38 items, and the cumulative contribution rate reached 69.073%. The analysis of the items contained in each factor is shown below, and the final EFA results are shown in Table 2.

**Table 2:** The results of the final EFA

Factors	Items	Component				
		1	2	3	4	5
Sense of Gain	Fulfilling Personal Value	0.675				
	Professional Pride	0.642				
	Job-Related Well-being	0.594				
Internal Organization Development	Knowledge Level	0.744				
	Department Setting	0.727				
	Software and Hardware Facilities	0.726				
	Regulatory Regime	0.710				
	Specialist Construction	0.703				
	Learning Atmosphere	0.690				
	Teaching and Scientific Research	0.683				
	Leading and Administrative Capacity	0.680				
	Personnel Quality	0.676				
	Services Scope	0.644				
	Learning Resources	0.577				
	Human Resource Allocation	0.570				
Remuneration and Development	Medical Insurance		0.795			
	Working Subsidy		0.789			
	Wage		0.782			
	Social Insurance and Housing Accumulation Fund		0.722			
	Working Bonus		0.721			
	Holidays Arrangements		0.705			
	Performance Assessment		0.674			
	Professional Title Promotion		0.534			
Condition of the City Where the PHCI Is Located	Location			0.760		
	Economics			0.725		
	Development			0.715		
	Culture and Customs			0.700		
	Environment			0.699		
	Transportation			0.688		
	Local Reputation of the PHCI			0.626		
Job Responsibilities	Workload				0.872	
	Working Intensity				0.856	
	Working Stress				0.851	
	Working Hours				0.786	
Family Support	Spouse					0.806
	Parents					0.783
	House					0.762
	Children					0.733

#### Sense of gain and internal organization development

The first factor contained 15 items, but these items reflected different connotations. Specifically, 12 items, such as the regulatory regime, software and hardware facilities, and scope of services, reflected the internal construction and development of a PHCI, while the remaining three items, including fulfilling personal value, professional pride, and job-related well-being, reflected the working feelings of medical personnel. In addition, in the first EFA, the three items and basic livelihood security constituted the sixth factor, whereas basic livelihood security was deleted because of factor loads above 0.4 on the two factors, causing the remaining three factors to be rotated to the first factor. Based on the meaning of the items, we split the first factor into two factors.

For the three items, medical personnel have a sacred responsibility to safeguard the health of and provide rehabilitation, treatment, prevention, and health-care services to the residents, and the medical personnel need considerable knowledge, training, and practice, or to obtain professional titles, which requires a great deal of time and effort [46] to be competent, so most of them have a high sense of professional pride which is closely related to the work quality, job satisfaction, and intention to leave [47–49]; they hope to show personal value in the work. In addition, job-related well-being is an important emotional reflection of employees at work. Positive emotions could help employees overcome difficulties and thrive [50], while negative emotions can lead to stress, depression, and anxiety. Moreover, job-related well-being is a key factor in attracting and retaining employees [51] and plays a vital role in employment choices [52]; therefore, the three items were important indicators reflecting the possibility of medical personnel seeking employment at PHCIs, and they were collectively referred to as ‘Sense of Gain’. This study took Sense of Gain as the dependent variable to explore the relationship between various factors and the employment intention of medical personnel. The remaining 13 factors were collectively referred to as ‘Internal Organization Development’. It has conclusively been shown that the organization’s internal development is critical to the turnover rate, happiness, job satisfaction, and burnout of medical personnel [53–55].

Based on the above arguments, the following hypothesis has been formulated:

##### Hypothesis 1 (H1)

*Internal Organization Development* has a positive effect on *Sense of Gain*.

#### Remuneration and development

This factor included 9 items, reflecting a focus on remuneration and individual development by seeking employment at PHCIs. Employee remuneration and individual development are undoubtedly influenced by organizational arrangements, systems, and operating conditions. Remuneration and benefits are also important dimensions of employee satisfaction [56]. Previous studies [57–59] have pointed out that because of low remuneration and poor individual development prospects, community health workers have low job satisfaction and enthusiasm and intend to leave their posts. At the same time, according to the growth of medical personnel [46], long-term pre-training and time investment make them hopeful about receiving good economic returns and achieving individual development in their work. Therefore, we propose that appropriate remuneration and individual development can enhance the sense of gain of medical personnel, which is also an important driving force for employment at PHCIs.

Based on the above arguments, the following hypothesis has been formulated:

##### Hypothesis 2 (H2)

*Internal Organization Development* has a positive effect on *Remuneration and Development*.

##### Hypothesis 3 (H3)

*Remuneration and Development* have a positive effect on *Sense of Gain*.

#### Condition of the City where the PHCI is located

There were 7 items in this factor, which reflected that medical personnel were concerned not only with internal organization development but also with the external environment of PHCIs. Studies have pointed out that people tend to work in places with a high level of economic development, a good environment, and convenient transportation [60, 61]. These areas have higher living and income standards and more opportunities for individual development, which would increase the well-being of workers in their lives and work. This is one of the important reasons for people from remote areas to move to larger cities.

Based on the above arguments, the following hypothesis has been formulated:

##### Hypothesis 4 (H4)

*Condition of the City Where the PHCI Is Located* has a positive effect on *Remuneration and Development*.

##### Hypothesis 5 (H5)

*Condition of the City Where the PHCI Is Located* has a positive effect on *Sense of Gain*.

#### Job responsibilities

This factor included five items, which reflected the concern of medical personnel about their specific work content and job responsibilities. Several studies [62–64] have documented that a strong correlation between job characteristics (such as job content and stress) and job-related happiness, and come up with strategies for improving employees’ job-related happiness from the perspective of job responsibility. Job-related happiness is closely related to career choice, so job responsibility is an important influencing factor for employees’ career choices. At the same time, workload, work time and responsibilities depend on the internal division of labor and management system of the organization. Proper division of labor, reasonable human resources management and management system will clarify the job responsibilities of employees, and the job responsibilities (workload, working time, and job stress) will become the promotion factor rather than the hindrance factor of job-related happiness [65, 66]. Therefore, we recommend that Internal Organizational Development influences Job Responsibility and that Job Responsibility influences Sense of Gain.

Based on the above arguments, the following hypotheses have been formulated:

##### Hypothesis 6 (H6)

*Internal Organization Development* has a positive effect on *Job Responsibilities.*

##### Hypothesis 7 (H7)

*Job Responsibilities* have a positive effect on *Sense of Gain*.

This study provides a new thinking direction for improving the well-being of employees in enterprises.

#### Family support

This factor included 4 items, which represent the family factors considered when medical personnel seek employment at a PHCI. Studies [67, 68] have pointed out that family support can relieve job stress and prevent negative job-related outcomes, such as job burnout. In addition, family members have also been shown to provide both instrumental and affective support and a positive impact on the working life of employees [69, 70]. A meta-analysis [71] showed that the conflict between work and family has a strong impact on the high turnover rate of medical personnel and that reducing this conflict can improve their happiness. In addition, communication research [72] has shown that the family as a socialization agent conveys both extrinsic and intrinsic work values for developing a professional identity. Therefore, we proposed that family support would have a positive impact on the professional identity and work enthusiasm of medical personnel. Besides, on the one hand, the income level of employees is closely related to parental support, children’s education and quality of life [73]. On the other hand, previous studies have demonstrated the existence of interrelated, co-facilitating mechanisms in individual and family development [74]. Finally, the life and work of family members would inevitably be affected by the Condition of the City Where the PHCI Is Located via the economy, culture, etc.

Based on the above arguments, the following hypotheses have been formulated:

##### Hypothesis 8 (H8)

*Condition of the City Where the PHCI Is Located* has a positive effect on *Family Support*.

##### Hypothesis 9 (H9)

*Remuneration and Development* has a positive effect on *Family Support.*

##### Hypothesis 10 (H10)

*Family Support* has a positive effect on *Sense of Gain*.

**Fig. 2:**
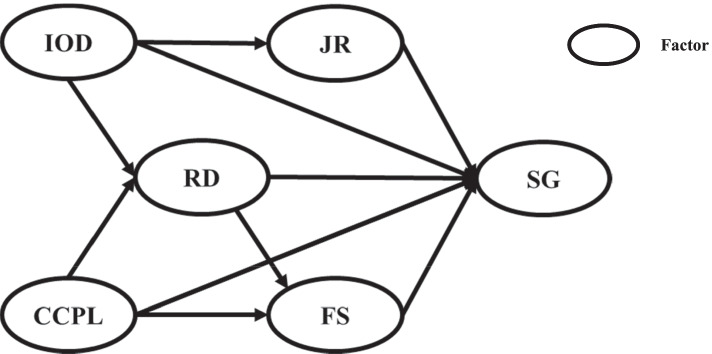
The hypothetical structural equation model. Note: SG, Sense of Gain; RD, Remuneration and Development; IOD, Internal Organization Development; CCPL, Condition of the City Where the PHCI Is Located; JR, Job Responsibilities; FS, Family Support

Based on the above hypothesis, we formulate our proposed relationships in a hypothesis structural model, which is shown in Fig. 2.

### Confirmatory factor analysis

CFA is the measurement part of SEM, which reflects the relationship between potential variables and their indicators. The reliability analysis, internal consistency reliability, convergent validity, and discriminant validity were assessed in the measurement model, and the results of CFA are shown in Table 3. In the process, position development and learning resources were removed because the standardized factor loadings were lower than 0.7 [75].

**Table 3:** Reliability and validity test

Factors	Items	Standardized factor loading	SMC	CR	AVE	Cronbach alpha
Condition of the City Where the PHCI Is Located	Development	0.861	0.741	0.933	0.665	0.932
	Economics	0.830	0.689			
	Environment	0.815	0.664			
	Transportation	0.815	0.664			
	Location	0.813	0.661			
	Culture and Customs	0.805	0.648			
	Local Reputation of the PHCI	0.767	0.588			
Remuneration and Development	Working Subsidy	0.899	0.808	0.942	0.699	0.941
	Medical Insurance	0.887	0.787			
	Working Bonus	0.861	0.741			
	Wage	0.856	0.733			
	Performance Assessment	0.824	0.679			
	Social Insurance and Housing Accumulation Fund	0.763	0.582			
	Holiday Arrangements	0.751	0.564			
Internal Organization Development	Specialist Construction	0.823	0.677	0.945	0.609	0.944
	Department Setting	0.817	0.668			
	Regulatory Regime	0.817	0.668			
	Human resource allocation	0.813	0.661			
	Knowledge Level	0.807	0.651			
	Software and Hardware Facilities	0.783	0.613			
	Personnel Quality	0.782	0.612			
	Learning Atmosphere	0.769	0.591			
	Teaching and scientific research	0.742	0.551			
	Services Scope	0.709	0.503			
	Leading and Administrative Capacity	0.709	0.503			
Job Responsibilities	Workload	0.920	0.846	0.910	0.717	0.907
	Working Intensity	0.887	0.787			
	Working Stress	0.830	0.689			
	Working Hours	0.740	0.548			
Family Support	House	0.813	0.661	0.865	0.615	0.864
	Parents	0.808	0.653			
	Spouse	0.787	0.619			
	Children	0.727	0.529			
Sense of Gain	Fulfilling Personal Value	0.865	0.748	0.855	0.664	0.851
	Job-Related Well-being	0.807	0.651			
	Professional Pride	0.769	0.591			

Cronbach’s alpha values were calculated for each factor to perform reliability analysis, and all the values were greater than 0.8, indicating high reliability. In addition, the composite reliability (CR) reflects the internal consistency reliability of the measurement model. The results showed that the CR values ranged from 0.855 to 0.945, indicating a high internal consistency among the constructs. The average variance extracted (AVE) and standardized factor loading reflects the extent to which a measure correlates positively with alternative measures of the same construct, named convergent validity [76]. The results showed that the AVE values were above 0.5 [77], and all the standardized factor loadings were greater than 0.7 [43], indicating good convergent validity. Discriminant validity [78] refers to the extent of which a construct is distinct from other constructs by empirical standards, and the criterion was that the square roots of the AVEs of each construct should be higher than its correlation with any other construct. As shown in Table 4, the square roots of the AVEs of each construct were highest, indicating that all constructs had adequate discriminant validity [79].

**Table 4:** Discriminant validity of constructs

Factors	SG	FS	JR	IOD	RD	CCPL
SG	**0.815**					
FS	0.514	**0.785**				
JR	0.467	0.503	**0.847**			
IOD	0.745	0.470	0.431	**0.780**		
RD	0.612	0.574	0.405	0.694	**0.836**	
CCPL	0.605	0.528	0.407	0.721	0.643	**0.816**

The bold diagonal data are the square root of the AVE and the others are latent variable correlations

*SG* Sense of Gain, *FS* Family Support, *JR* Job Responsibilities, *IOD* Internal Organization Development, *RD* Remuneration and Development, *CCPL* Condition of the City Where the PHCI Is Located

Therefore, we concluded that the measurement model has good reliability and validity to test the structural model of our proposed hypotheses.

Common method variance (CMV) refers to the amount of spurious covariance shared among variables because of the common method used in collecting data [80], so we tested the possibility for CMV through the three criteria. First, as shown in Table 4, the correlation coefficients between the structures were less than 0.9, indicating that there were no pairs with strong correlations [43]. Second, the Harman single-factor test was conducted by PCA, and the results showed that the first extracted factor in the unrotated solution accounted for 45.691% of the variance, which was less than 50% [81]. Finally, the marker variable technique [82, 83] was applied to test the variance, and health condition, which was not relevant for our research, was set as a marker variable. The correlation coefficients between the health condition and other variables ranged from − 0.147 to 0.052, so common method variance was ruled out in our research.

### Structural model analysis

The t-values and *p*-values of each path were computed in AMOS to test the hypothesized relationships. The results showed that Internal Organization Development (*β* = 0.558; *P* < 0.001), Remuneration and Development (*β* = 0.078; *P* = 0.039), Job Responsibilities (*β* = 0.112; *P* < 0.001) and Family Support (*β* = 0.127; *P* < 0.001) had a significantly positive effect on the Sense of Gain of medical personnel seeking employment at PHCIs, so H1, H3, H7, and H10 were supported. However, the hypothesized relationship between the Condition of the City Where the PHCI Is Located and Sense of Gain (*β* = 0.043; *P* = 0.261) was not significant, so H4 was not supported. In addition, both Internal Organization Development (*β* = 0.479; *P* < 0.001) and the Condition of the City Where the PHCI Is Located (*β* = 0.312; *P* < 0.001) had significantly positive effects on Remuneration and Development, so H2 and H4 were supported. Internal Organization Development had a significantly positive effect on Job Responsibilities (*β* = 0.446; *P* < 0.001), and the Condition of the City Where the PHCI Is Located and Remuneration and Development had a significantly positive effect on Family Support (*β* = 0.271, *P* < 0.001; *β* = 0.397, *P* < 0.001;), indicating that H6, H8 and H9 were supported.

**Fig. 3:**
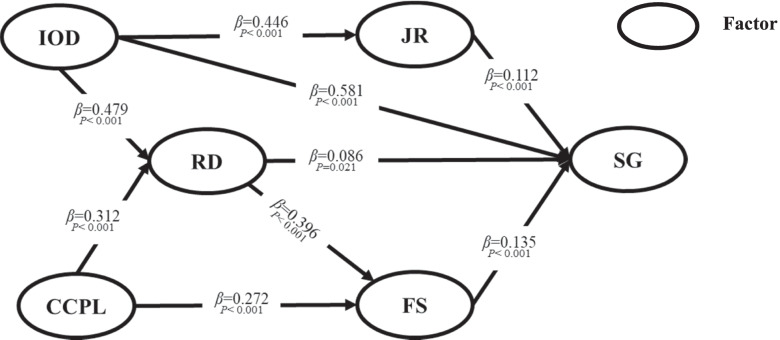
The results of the modified structural equation model. Note: SG, Sense of Gain; RD, Remuneration and Development; IOD, Internal Organization Development; CCPL, Condition of the City Where the PHCI Is Located; JR, Job Responsibilities; FS, Family Support

After the insignificant path was removed, the modified model was refitted. The results of the modified structural equation model are demonstrated in Fig. 3, and the coefficients of the supported hypothesis paths changed slightly. The effect of Job Responsibilities, Internal Organization Development, Remuneration and Development and Family Support on Sense of Gain was 0.112,0.581,0.086 and 0.135 respectively. Meanwhile, the effect of Internal Organization Development on Job Responsibilities was 0.446, and that of Condition of the City Where the PHCI Is Located on Family Support is 0.272. In the last, the effect of internal and external construction of PHCIs on Remuneration and Development is 0.479 and 0.312 respectively.

The fit of the modified model is shown in Model 1 in Table 5, and the values indicated an acceptable-fitting model. The validation results for model stability in the third stage are shown in Model 2 of Table 5, and the results show that the model is acceptable.

**Table 5:** The fit of the structural equation model

Model	SRMR	RMSEA	CFI	TLI	IFI	NFI
Reference	< 0.1	< 0.08	> 0.9	> 0.9	> 0.9	> 0.9
Model 1	0.056	0.06	0.929	0.923	0.929	0.914
Model 2	0.073	0.061	0.944	0.91	0.944	0.941

Model 1 = The modified model established in the second phase, Model 2 = The model established in the third phase

In addition, in the second and third phases, the bootstrapping technique in AMOS was applied to explore the mediating role of Family Support, Job Responsibilities, and Remuneration and Development. The indirect effects were obtained with 5000 bootstraps resamples. The results showed that Job Responsibilities and Remuneration and Development significantly mediated the relationship between Internal Organization Development and Sense of Gain, while Family Support and Remuneration and Development also played a significant mediating role from the Condition of the City Where the PHCI Is Located to Sense of Gain. Table 6 shows the direct and indirect effects of each factor in the two phases. Internal Organization Development had the greatest effect on the Sense of Gain (0.698 and 0.522, respectively), followed by Family Support (0.135 and 0.328, respectively). While the direct effect of the Condition of the City Where the PHCI Is Located was not significant, there was a significant indirect effect on the Sense of Gain through Remuneration and Development and Family Support (0.080 and 0.172, respectively).

**Table 6:** Results of direct, indirect and total effects of each factor

Path	Effect	Model 1			Model 2	
		Coefficient	*P*		Coefficient	*P*
IOD → JR	Direct	0.446	<0.001	Direct	0.499	<0.001
IOD → RD	Direct	0.479	<0.001	Direct	0.452	<0.001
IOD → FS	Indirect	0.190	<0.001	Indirect	0.140	<0.001
IOD → SG	Direct	0.581	<0.001	Direct	0.401	<0.001
	Indirect	0.117	<0.001	Indirect	0.121	<0.001
	Total	0.698	<0.001	Total	0.522	<0.001
CCPL→ FS	Direct	0.272	<0.001	Direct	0.332	<0.001
	Indirect	0.123	<0.001	Indirect	0.124	<0.001
	Total	0.395	<0.001	Total	0.457	<0.001
CCPL→ RD	Direct	0.312	<0.001	Direct	0.401	<0.001
CCPL→ SG	Indirect	0.080	<0.001	Indirect	0.172	<0.001
RD → SG	Direct	0.086	0.021	Direct	0.054	0.006
	Indirect	0.053	<0.001	Indirect	0.102	<0.001
	Total	0.139	<0.001	Total	0.156	<0.001
RD → FS	Direct	0.396	<0.001	Direct	0.31	<0.001
JR → SG	Direct	0.112	<0.001	Direct	0.101	<0.001
FS → SG	Direct	0.135	<0.001	Direct	0.328	<0.001

*SG* Sense of Gain, *FS* Family Support, *JR* Job Responsibilities, *IOD* Internal Organization Development, *RD* Remuneration and Development, *CCPL* Condition of the City Where the PHCI Is Located. Model 1 = The model established in the second phase, Model 2 = The established in the third phase

## Discussion

This study explored the factors that influence medical personnel in urban public hospitals seeking employment at PMCHIs from the perspective of the target group based on implicit theory and the lexical approach. Through pre-investigation and a three-phase investigation, the influences of six factors, including Sense of Gain, Internal Organization Development, Remuneration and Development, Job Responsibility, Condition of the City Where the PHCI Is Located, and Family Support, on the willingness of medical personnel to seek employment at PHCIs and the corresponding action path were clarified. Based on the fit and verification of the model, we propose that the model is acceptable and credible. Therefore, this study provides a theoretical perspective for guiding medical personnel to seek employment at PHCIs, improving human resource development in PHCIs, and promoting the utilization of primary health care services.

Remuneration and development positively affected the willingness of medical personnel to seek employment at PHCIs, which was related not only to the common needs of job-choosing groups but also to the current situation of PHCIs. Previous studies have shown that income level and income satisfaction were important factors affecting the turnover and career choice of primary-level doctors [84, 85], which is consistent with our findings. In China, because of the low technical level and underdeveloped environmental facilities of PHCIs, patients have a low level of acceptance and willingness to seek medical treatment at PHCIs [86–88]. As a result, PHCIs only provide basic public health services and a small number of medical services, and the income of medical personnel mainly comes from government financial subsidies. Therefore, remuneration and personal development are limited, which results in low enthusiasm and a serious loss of medical personnel. In turn, the medical service of PHCIs could not be improved, and patient’s willingness to seek medical treatment would decrease again. Therefore, from the perspective of medical personnel, it is necessary to improve remuneration to a certain extent and then to improve enthusiasm about working in PHCIs. In addition, differentiated medical insurance policies and hierarchical diagnosis and treatment systems should be formulated to encourage patients to seek medical treatment in PHCIs. Only through the joint change of the two sides can remuneration and development be improved, and the willingness of medical personnel to seek employment at PHCIs will be increased.

Family support had a positive effect on the willingness of medical personnel to seek employment at PHCIs, indicating that employees considered the changes in the work and life of their family members when choosing a job. Previous studies have also shown a negative correlation between family support and turnover intention [89, 90]. In China, family is the core component of a person’s life and affects people’s work and development. Therefore, we propose that providing suitable living and working conditions for family members of medical personnel will facilitate the willingness of medical personnel to seek employment at PHCIs.

The degree of medical personnel’s attention to job responsibility had a positive effect on the willingness of medical personnel to seek employment at PHCIs. Prior studies have noted that workload, work intensity, and work pressure are important factors influencing the satisfaction and turnover intention of medical personnel in PHCIs [53, 91, 92]. Against the background of the implementation of the hierarchical diagnosis and treatment system in China’s new medical reform, PHCIs are responsible for the diagnosis and treatment of common and frequently occurring diseases, management of chronic diseases, and rehabilitation treatment in the congruency period. In addition, scholars suggest that under the background of COVID-19, the medical personnel in PHCIs for the prevention and control of the epidemic plays an important role [93, 94]. Therefore, medical personnel in PHCIs are the people who have the earliest and closest contact with patients, and they are the gatekeepers of residents’ health. At the same time, the role of medical personnel in PHCIs has expanded in the context of the integration of public health services and clinical services [95, 96], so they are under considerable work pressure. Therefore, we propose that the willingness of medical personnel to seek employment at PHCIs can be improved by matching the responsibilities with their interests, such as providing appropriate remuneration according to the work content and balancing the pressure from the job.

Internal Organization Development had a significant and important direct and indirect effect on the willingness of medical personnel to seek employment at PHCIs. Previous studies have also shown that teamwork environment, leadership behavior and management system affect employees’ job satisfaction turnover intention [97, 98]. On the one hand, as a member of a PHCI, the work of medical personnel is directly affected by the organization’s construction and management, which is an important part of the medical personnel’s work experience. On the other hand, the internal construction of the organization affects remuneration and development, as well as job responsibility, which are the factors that affect work enthusiasm and job-related well-being. In addition, we propose that the internal construction of the organization affects patients’ attitudes toward PHCIs and their willingness to seek medical services in PHCIs, thus affecting the work of medical personnel and the development of PHCIs. Therefore, scientific and reasonable management systems, good working facilities, and environments will increase the sense of well-being and belonging and improve the enthusiasm of medical personnel. Regarding human resource development in PMHCs, the improvement of internal organization construction will increase the willingness of medical personnel to seek employment at PHCIs.

The Condition of the City Where the PHCI Is Located had no significant direct effect on the willingness of medical personnel to seek employment at PHCIs. The reason may be that medical personnel are more concerned with proximal factors such as individuals, family, and internal development than with the external environment. However, the results showed that the Condition of the City Where the PHCI Is Located had a significant effect on the family members and remuneration and development of medical personnel. In addition, the results of the mediation effects showed that the external environment, through Remuneration and Development and Family Support, has a significant effect on Sense of Gain. Therefore, we conclude that the Condition of the City Where the PHCI Is Located has an indirect and important effect on the willingness of medical personnel to seek employment at PHCIs.

### Strengths and limitations of this study

The major strengths of this research include the following aspects. First, the analysis explored the factors from the perspective of the guided objects rather than the government and PHCIs, making up for the lack of understanding of the actual needs of medical personnel. Second, this research analyzed the effects of the internal and external environment of PHCIs, family, job responsibility, remuneration, and development on the willingness of medical personnel to seek employment at PHCIs, and the factors discussed were comprehensive and representative. Third, this research established the influence model of medical personnel seeking employment at PHCIs through pre-investigation and three-phase investigation. The process was complete and rigorous, and the resulting model was representative and stable. However, two limitations of the research also need to be acknowledged.

First, because of the large differences in different regions of China, this research cannot fully reflect the situation in all regions, so further analysis based on local conditions is needed. Second, the sampling methods and the changing subjects in the investigation may affect the accuracy of the results, whereas we communicated with experts and local administration leaders to ensure that the samples accurately reflected the overall situation.

## Conclusions

This research explored the factors that affected medical personnel to seek employment at PHCIs. We conclude that the main factors include the sense of gain, family support, remuneration and development, job responsibility and internal and external construction of PHCIs. The improvement of family support, remuneration, individual development, and reasonable work arrangement can improve the medical personnel’s sense of gain to seek employment at PHCIs. In addition, internal organizational development has a direct and indirect positive effect on the sense of gain through job responsibility, remuneration and development, whereas the external environment of PHCIs has a positive and indirect effect on sense of gain through remuneration and development and family support. Therefore, the development of PHC providers can be promoted by formulating incentive policies, for example, paying attention to the family members of medical personnel, establishing a reasonable remuneration system, providing suitable development opportunities, arranging work rationally and improving the internal construction of PHCIs. This research contributes theoretical and practical support for human resource development in PHC and promotes equity in health.

## Data Availability

The datasets used and analysed during the current study are available from the corresponding author on reasonable request.

## Abbreviations

PHCIs: Primary health care institutions
SG: Sense of Gain
RD: Remuneration and Development
IOD: Internal Organization Development
CCPL: Condition of the City Where the PHCI Is Located
JR: Job Responsibilities
FS: Family Support
AVE: Average variance extracted
EFA: Exploratory factor analysis
CFA: Confirmatory factor analysis
SEM: Structural equation modeling
VR: Varimax rotation
PCA: Principal component analysis
RMSEA: The root mean square error of approximation
CFI: The comparative fit index
TLI: The Tucker-Lewis index
IFI: The incremental fit index

## Footnotes

1 In China, the medical service system is mainly divided into hospitals, professional public health institutions and primary health care institutions. The hospital’s function lies in the provision of medical service, the diagnosis and treatment of critical and difficult diseases, as well as personnel training and medical scientific research. Professional public health institutions provide professional public health service, including disease prevention and control, health education, maternal and child health care, mental health, first aid, blood collection and supply, comprehensive supervision and enforcement, food safety risk assessment and standard management, family planning, birth defect prevention and treatment, etc. The primary health care institutions are mainly responsible for providing basic services such as prevention, health care, health education and family planning, as well as diagnosis and treatment services for common and frequently encountered diseases, and rehabilitation, nursing services and referral services for some diseases.
